# Nickel chloride (NiCl_2_)-caused inflammatory responses *via* activation of NF-κB pathway and reduction of anti-inflammatory mediator expression in the kidney

**DOI:** 10.18632/oncotarget.5759

**Published:** 2015-09-21

**Authors:** Hongrui Guo, Huidan Deng, Hengmin Cui, Xi Peng, Jing Fang, Zhicai Zuo, Junliang Deng, Xun Wang, Bangyuan Wu, Kejie Chen

**Affiliations:** ^1^ Key Laboratory of Animal Diseases and Environmental Hazards of Sichuan Province, Sichuan Agricultural University, Ya'an, Sichuan, China; ^2^ College of Veterinary Medicine, Sichuan Agricultural University Ya'an, Sichuan, China

**Keywords:** NiCl, inflammation, NF-κB, mRNA expression, kidney, Immunology section

## Abstract

Nickel (Ni) or Ni compounds target a number of organs and produce multiple toxic effects. Kidney is the major organ for Ni accumulation and excretion. There are no investigations on the Ni- or Ni compounds-induced renal inflammatory responses in human beings and animals at present. Therefore, we determined NiCl_2_-caused alteration of inflammatory mediators, and functional damage in the broiler's kidney by the methods of biochemistry, immunohistochemistry and quantitative real-time polymerase chain reaction (qRT-PCR). Dietary NiCl_2_ in excess of 300 mg/kg caused the renal inflammatory responses that characterized by increasing mRNA expression levels of the pro-inflammatory mediators including tumor necrosis factor-α (TNF-α), cyclooxygenase-2 (COX-2), interleukin-1β (IL-1β), interleukin-6 (IL-6), interleukin-8 (IL-8) and interleukin-18 (IL-18) via the activation of nucleic factor κB (NF-κB), and decreasing mRNA expression levels of the anti-inflammatory mediators including interleukin-2 (IL-2), interleukin-4 (IL-4) and interleukin-13 (IL-13). Concurrently, NiCl_2_ caused degeneration, necrosis and apoptosis of the tubular cells, which was consistent with the alteration of renal function parameters including elevated alkaline phosphatase (AKP) activity, and reduced activities of sodium-potassium adenosine triphosphatase (Na^+^/K^+^-ATPase), calcium adenosine triphosphatase (Ca^2+^-ATPase), lactic dehydrogenase (LDH), succinate dehydrogenase (SDH) and acid phosphatase (ACP) in the kidney. The above-mentioned results present that the activation of NF-κB pathway and reduction of anti-inflammatory mediator expression are main mechanisms of NiCl2-caused renal inflammatory responses and that the renal function is decreased or impaired after NiCl2-treated.

## INTRODUCTION

Nickel (Ni) is a metal of widespread distribution in the environment. Also, Ni is a nutritionally essential trace metal for several animal species, micro-organisms and plants, and therefore either deficiency or toxicity symptoms can occur when too little or too much Ni is taken up [1]. Ni is important in the biological system, such as in enzyme activity and hormonal control, and also in RNA/DNA structure or function [2]. At present, Ni and Ni compounds, as essential constituents, have many industrial and commercial uses, and the progress of industrialization has led to increase pollutants into ecosystems [1, 3].

Ni is considered to be potentially hazardous to living organisms due to its genotoxicity, immunotoxicity, mutagenicity and carcinogenicity [4, 5]. The main exposure routes of human beings is inhalation, ingestion or dermal contact at occupational settings [6]. It has demonstrated that many forms of Ni can induce carcinoma formation in human beings and animals [7, 8]. Zheng et al. [9] have reported that nickel sulfate (NiSO_4_) induces oxidative stress and apoptosis in *Carassius auratus* liver. Nickel oxide nanoparticles (NiONPs) and NiSO_4_ can cause pulmonary inflammation and significant increase of interleukin-6 (IL-6) and interleukin-8 (IL-8) protein expression levels after 24 h treatment [10-12]. In previous studies, nickel chloride (NiCl_2_) elevates the protein expression of nucleic factor κB (NF-κB) and cyclooxygenase-2 (COX-2) in Beas-2B Cells [13]. NF-κB pathway is one of the most important regulators of inflammation. NF-κB can stimulate synthesis of pro-inflammatory mediators, such as tumor necrosis factor-α (TNF-α), COX-2, inducible nitric oxide synthase (iNOS), interleukin-1β (IL-1β), IL-6, and IL-8 [14]. NiONPs increases the protein expression of macrophage inflammatory protein-1α (MIP-1α), monocyte chemotactic protein-1 (MCP-1), interleukin-1α (IL-1α) and IL-1β in the lung of rats [15]. It has been reported that dietary supplemented with NiCl_2_ above 300 mg/kg is toxic to 3-wk-old male chicks [16]. Our previous studies have shown that dietary NiCl_2_ in excess of 300 mg/kg can cause immunotoxicity, oxidative damage and apoptosis in the kidney, spleen, small intestines and cecal tonsil of broilers [17-20].

Some studies have shown that the kidney serves as a major organ of Ni excretion and is a target organ for Ni toxicity due to Ni accumulation [21-23]. At present, there are no investigations on the Ni- or Ni compounds-induced renal inflammatory responses in human beings and animals. Therefore, the mRNA expression levels of inflammation-related genes, including NF-κB, TNF-α, COX-2, IL-1β, IL-6, IL-8, interleukin-18 (IL-18), and interleukin-2 (IL-2), interleukin-4 (IL-4) and interleukin-13 (IL-13) were detected by qRT-PCR and the protein expression of NF-κB was determined by immunohistochemistry in the present study. In order to monitor renal function, the histopathological changes and activities of sodium-potassium adenosine triphosphatase (Na^+^/K^+^-ATPase), calcium adenosine triphosphatase (Ca^2+^-ATPase), lactic dehydrogenase (LDH), succinate dehydrogenase (SDH), alkaline phosphatase (AKP), and acid phosphatase (ACP) in the kidney were also measured by biochemistry and experimental pathology.

## RESULTS

### Histopathological changes in the kidney

**Figure 1:**
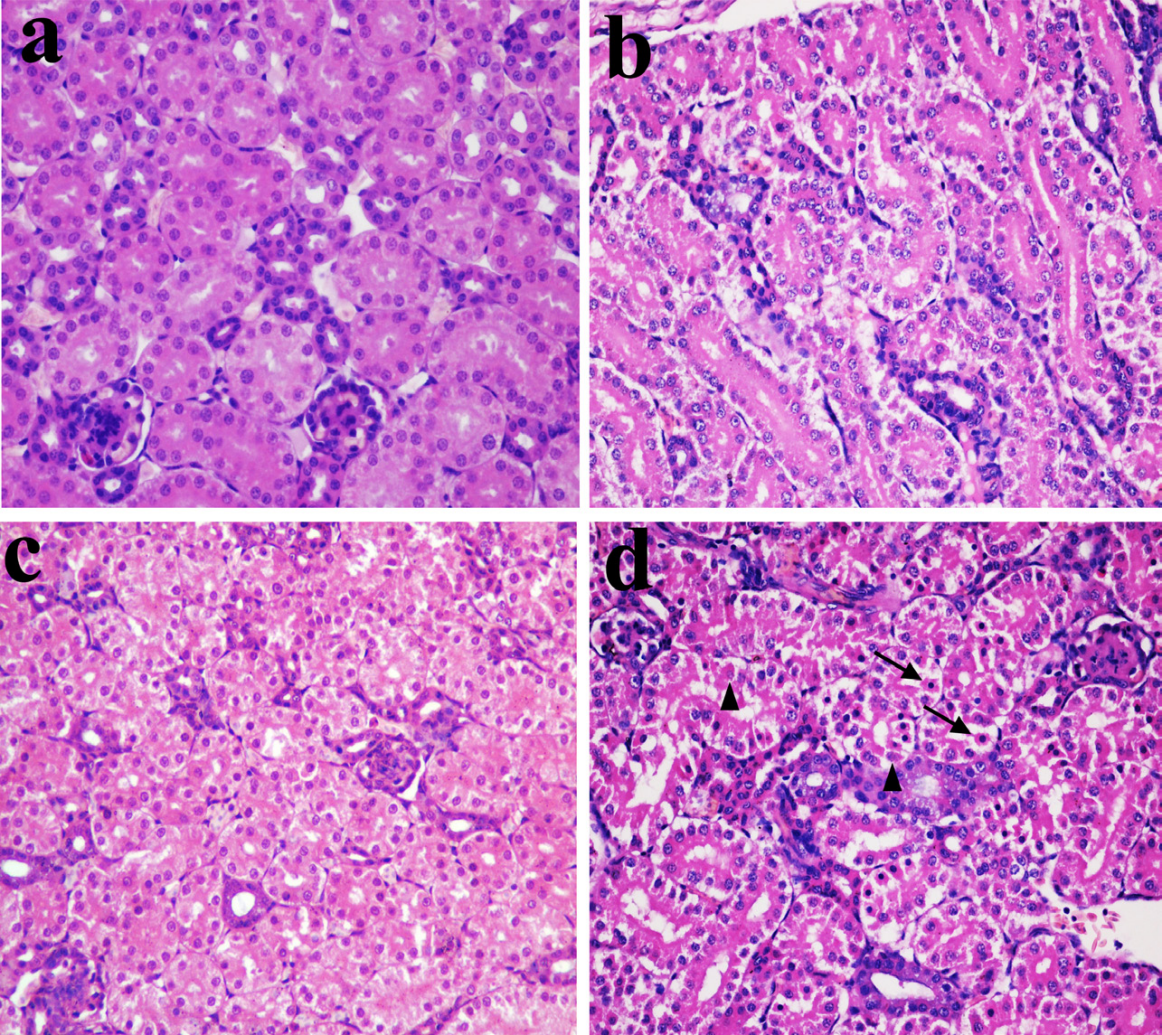
Histopathological changes in the kidney at 14 days of age **a.** Control group. No changes are observed (H•E ×400). **b.** 300 mg/kg group. Tubular cells show slight granular degeneration (H•E ×400). **c.** 600 mg/kg group. Tubular cells show granular degeneration and vacuolar degeneration (H•E ×400). **d.** 900 mg/kg group. Tubular cells show obvious granular and vacuolar degeneration. Also, few necrotic tubular cells (▲) and apoptotic tubular cells (↑) are observed (H•E ×400).

**Figure 2:**
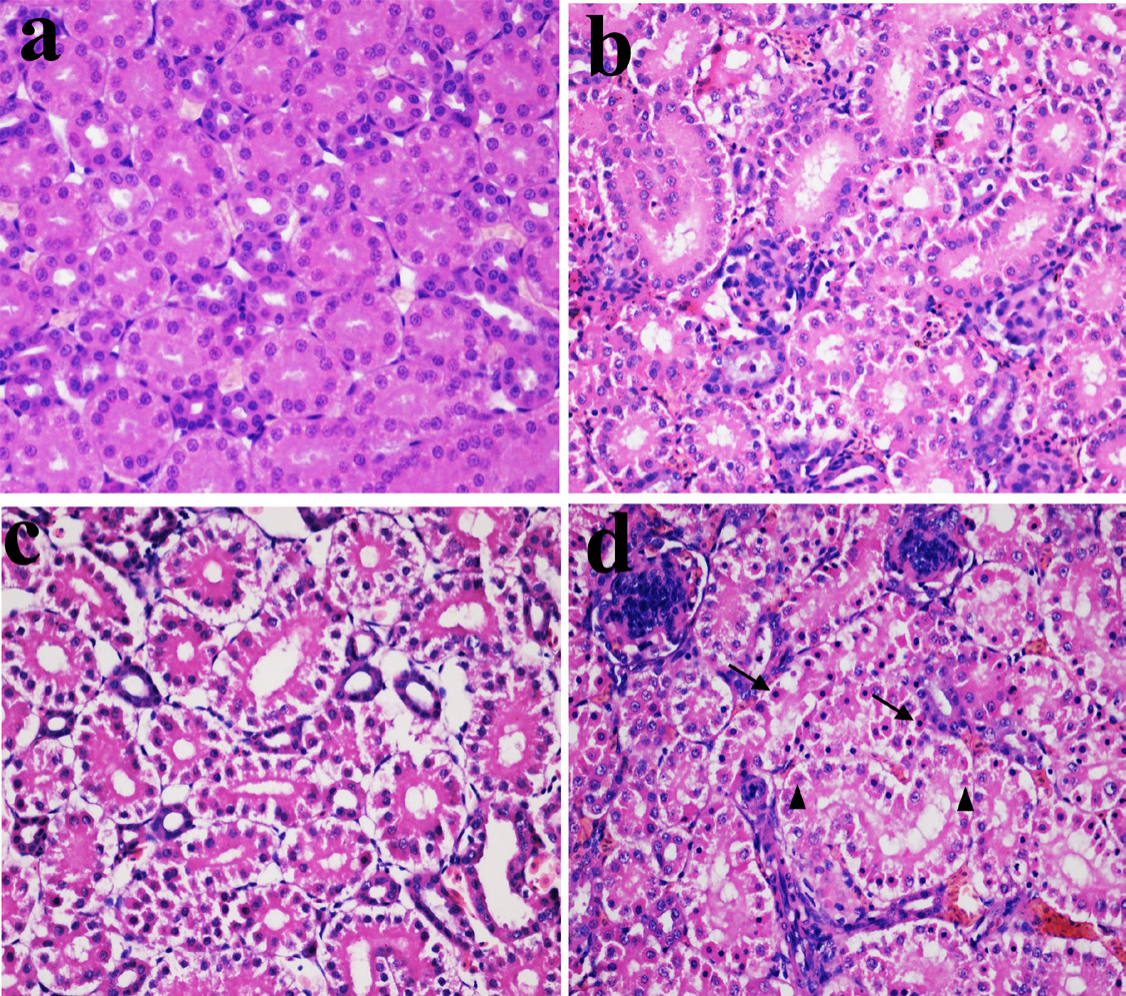
Histopathological changes in the kidney at 28 days of age **a.** Control group. No changes are observed (H•E ×400). **b.** 300 mg/kg group. Tubular cells show granular degeneration (H•E ×400). **c.** 600 mg/kg group. Tubular cells show obvious granular and vacuolar degeneration. Also, few necrotic tubular cells and apoptotic tubular cells are observed (H•E ×400). **d.** 900 mg/kg group. Tubular cells show marked granular and vacuolar degeneration. Also, some necrotic tubular cells (▲) and apoptotic tubular cells (↑) are observed (H•E ×400).

**Figure 3:**
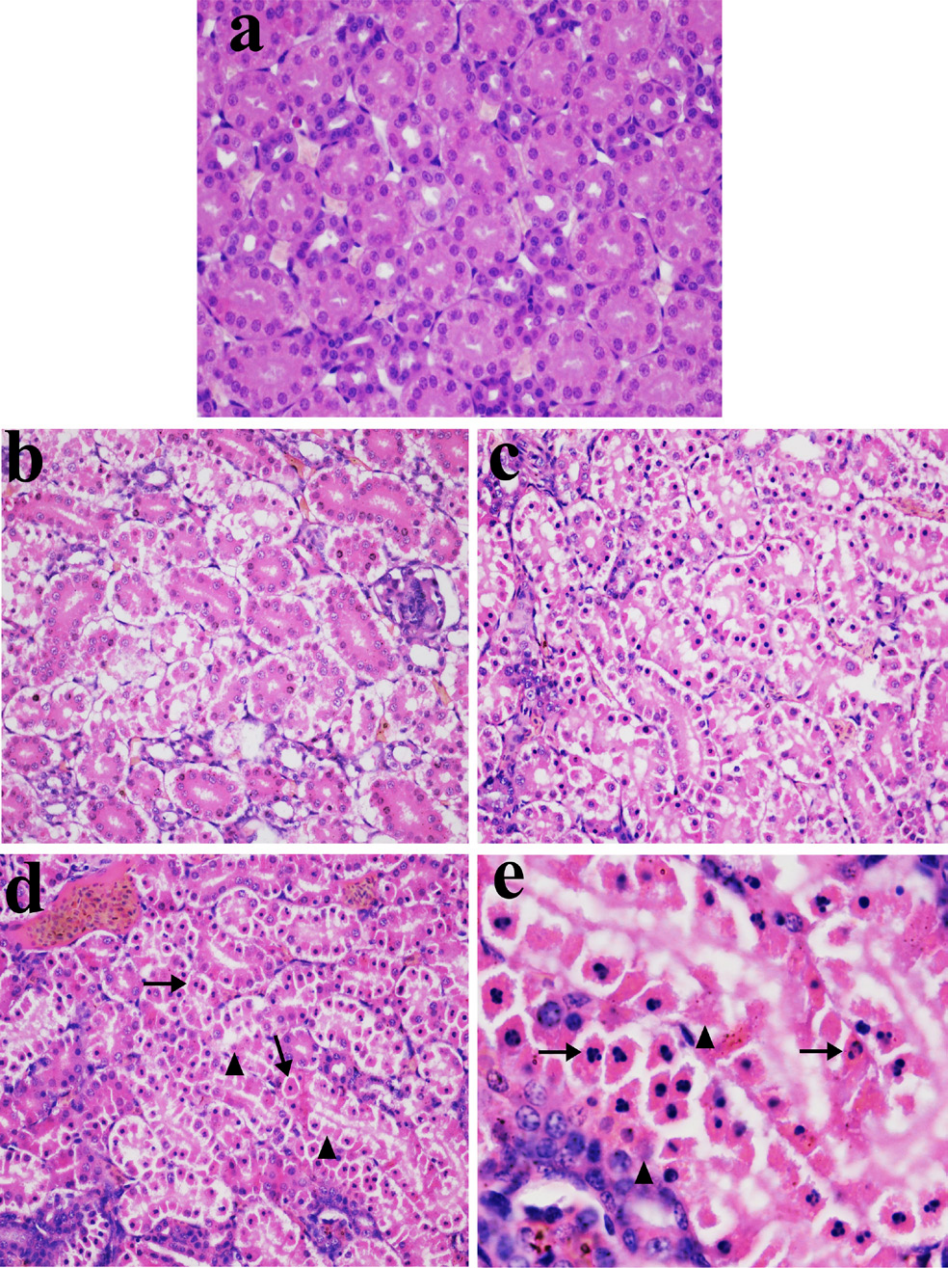
Histopathological changes in kidney at 28 days of age **a.** Control group. No changes are observed (H•E ×400). **b.** 300 mg/kg group. Tubular cells show granular and vacuolar degeneration (H•E ×400). **c.** 600 mg/kg group. Tubular cells show marked granular and vacuolar degeneration. Also, some necrotic tubular cells and apoptotic tubular cells are observed (H•E ×400). **d.** 900 mg/kg group. A large number of necrotic tubular cells (▲) and apoptotic tubular cells (↑) are observed (H•E ×400). **e.** 900 mg/kg group. Apoptotic bodies (↑), and the karyorrhexis, karyolysis and hypochromatosis of the necrotic tubular cells (▲) are observed (H•E ×1000).

In Figures 1, 2 and 3, NiCl_2_ resulted in does- and time-dependent histopathological changes in the kidney, including tubular granular degeneration, vacuolar degeneration, necrosis and apoptosis. In the granular and vacuolar degenerated tubular cells, tiny particles and small or large vacuoles were appeared in the cytoplasm. Karyorrhexis, karyolysis and hypochromatosis were appeared in the necrotic cells. In the apoptotic cells, cytoplasm was intensely eosinophilic, and nuclei were shrunken, dense, ring-shaped and crescentic. Apoptotic bodies were also observed.

### Changes of NF-κB expression levels in the kidney

**Figure 4:**
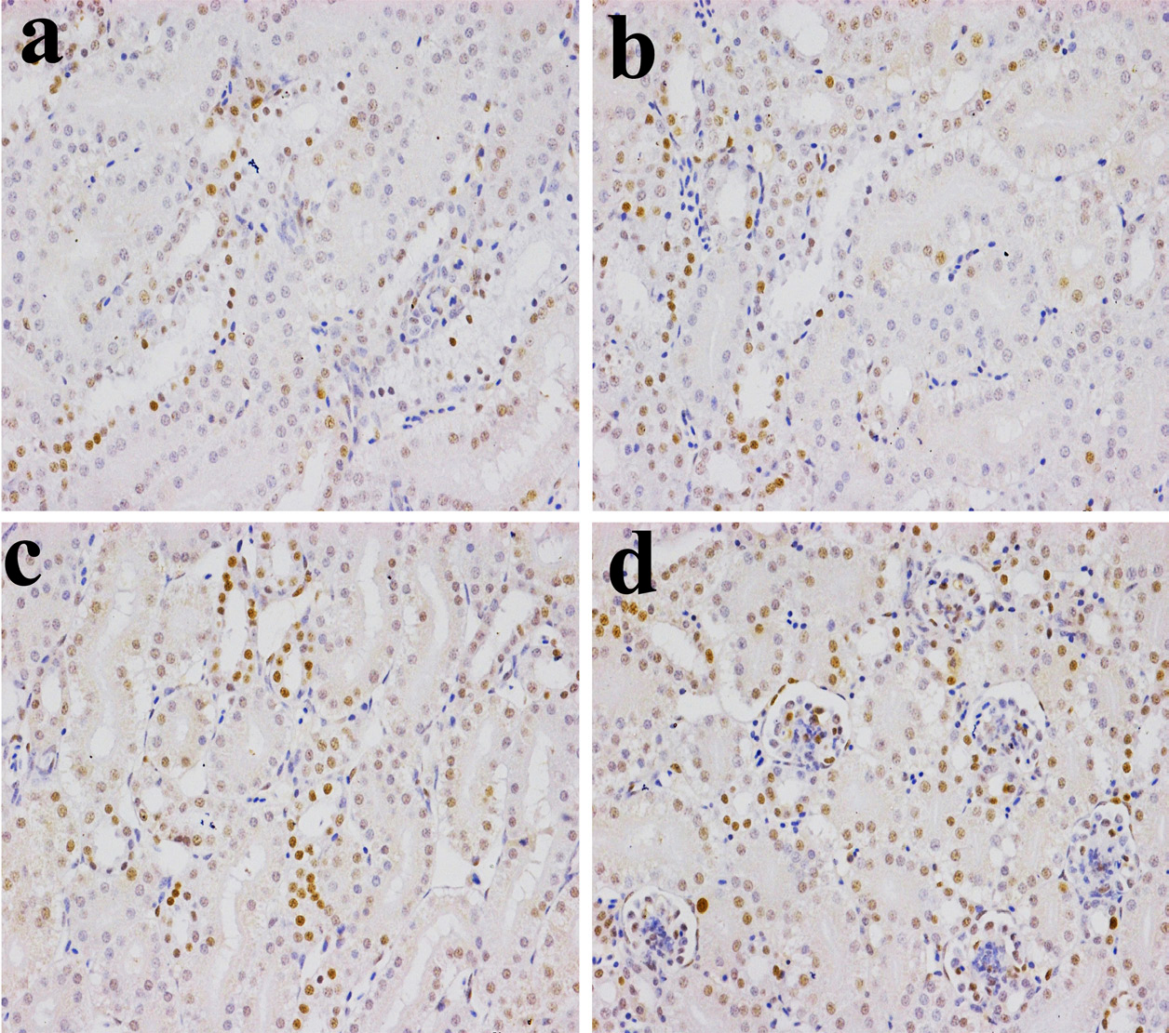
Changes of NF-κB protein expression levels in the kidney (×400) by immunohistochemistry at 14 days of age **a.** Control group. The NF-κB protein is less expressed. **b.** 300 mg/kg group. The NF-κB protein expression levels were no significantly changes than those in the control group. **c.** 600 mg/kg group. The NF-κB protein expression levels were significantly increased (*P* < 0.05) when compared with those in the control group. **d.** 900 mg/kg group. The NF-κB protein expression levels were significantly higher (*P* < 0.01) than those in the control group.

**Figure 5:**
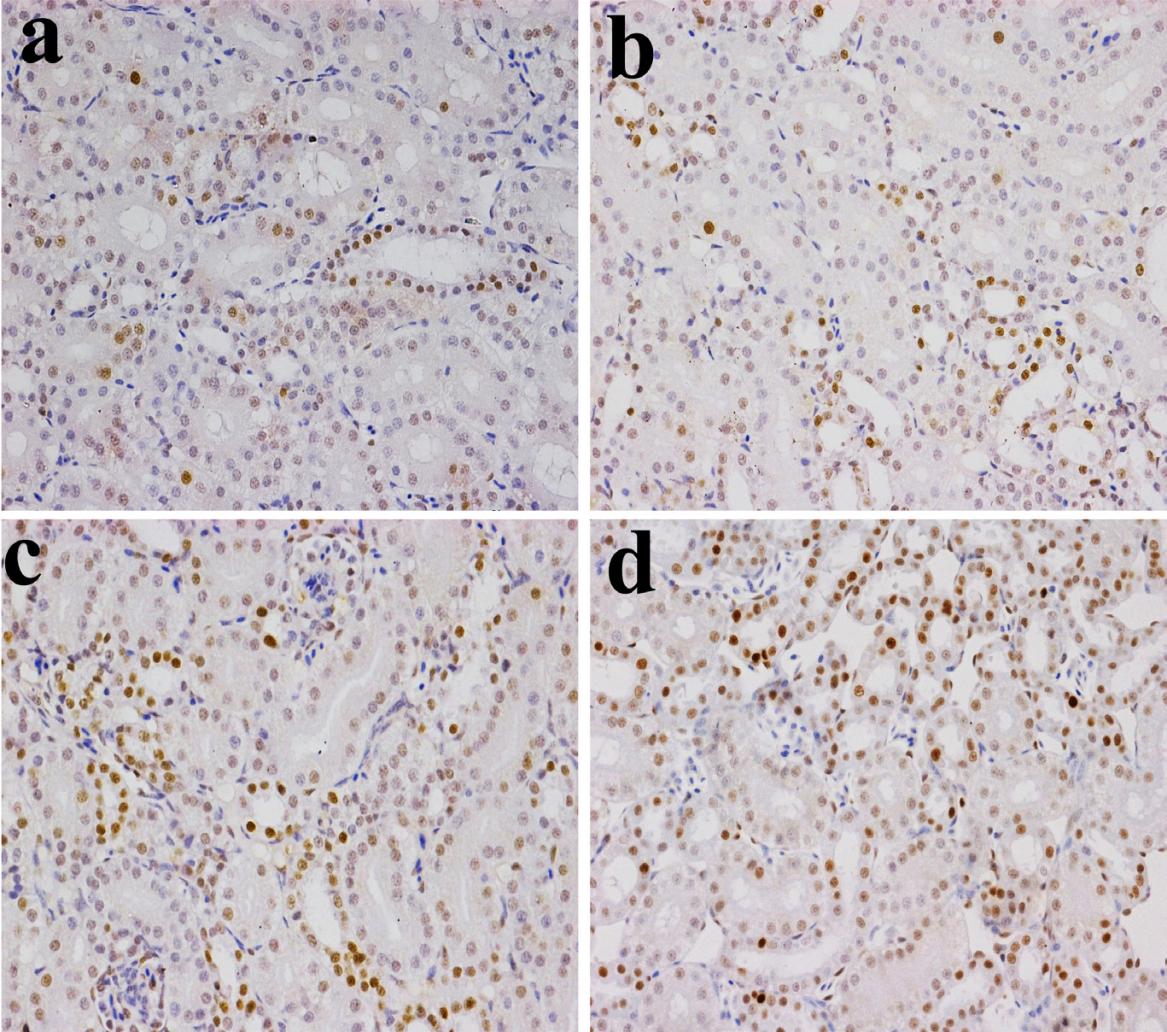
Changes of NF-κB protein expression levels in the kidney (×400) by immunohistochemistry at 28 days of age **a.** Control group. The NF-κB protein is less expressed. **b.** 300 mg/kg group. The NF-κB protein expression levels were no significantly changes than those in the control group. **c.** 600 mg/kg group. The NF-κB protein expression levels were significantly increased (*P* < 0.05) when compared with those in the control group. **d.** 900 mg/kg group. The NF-κB protein expression levels were significantly higher (*P* < 0.01) than those in the control group.

**Figure 6:**
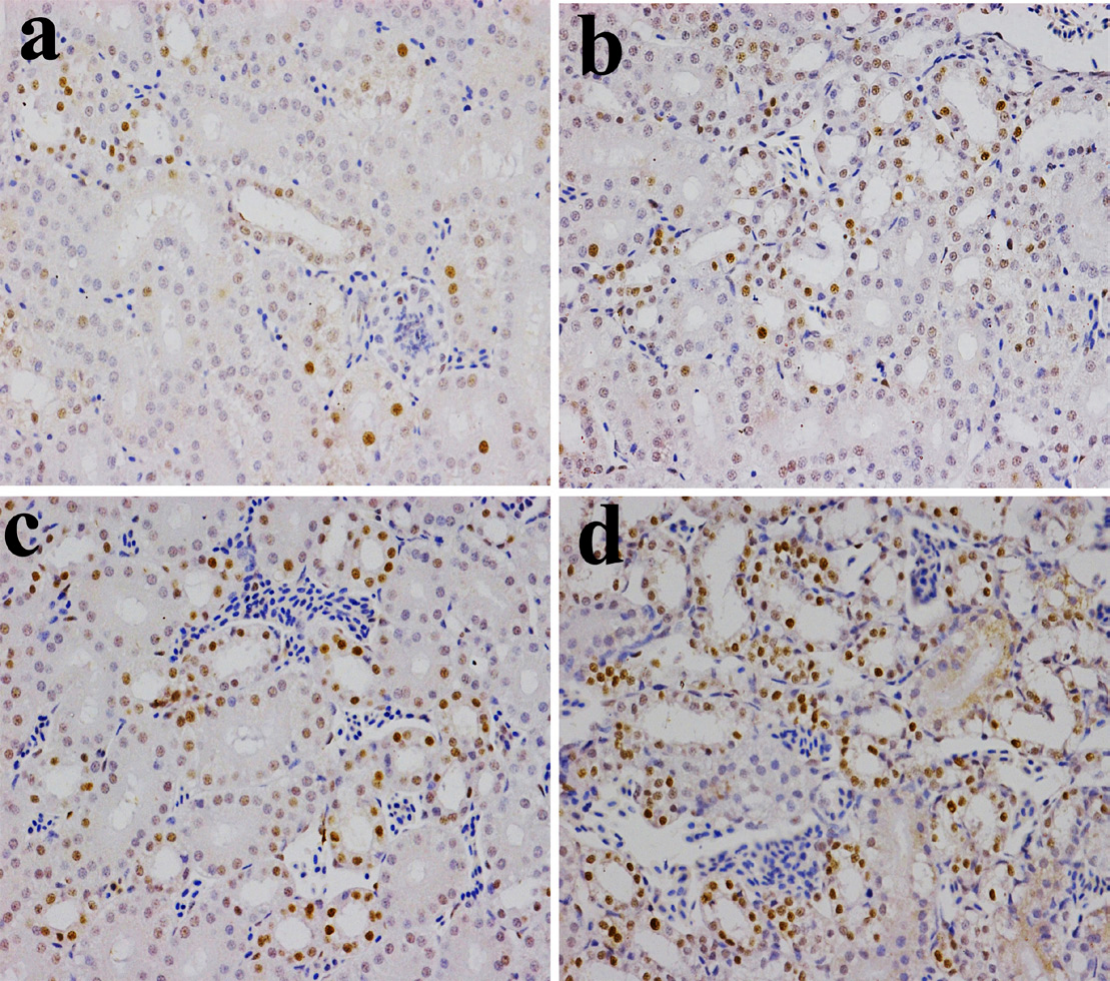
Changes of NF-κB protein expression levels in the kidney (×400) by immunohistochemistry at 42 days of age **a.** Control group. The NF-κB protein is less expressed. **b.** 300 mg/kg group. The NF-κB protein expression levels were significantly higher (*P* < 0.05) than those in the control group. **c.** 600 mg/kg group. The NF-κB protein expression levels were significantly increased (*P* < 0.01) when compared with those in the control group. **d.** 900 mg/kg group. The NF-κB protein expression levels were significantly higher (*P* < 0.01) than those in the control group.

**Figure 7:**
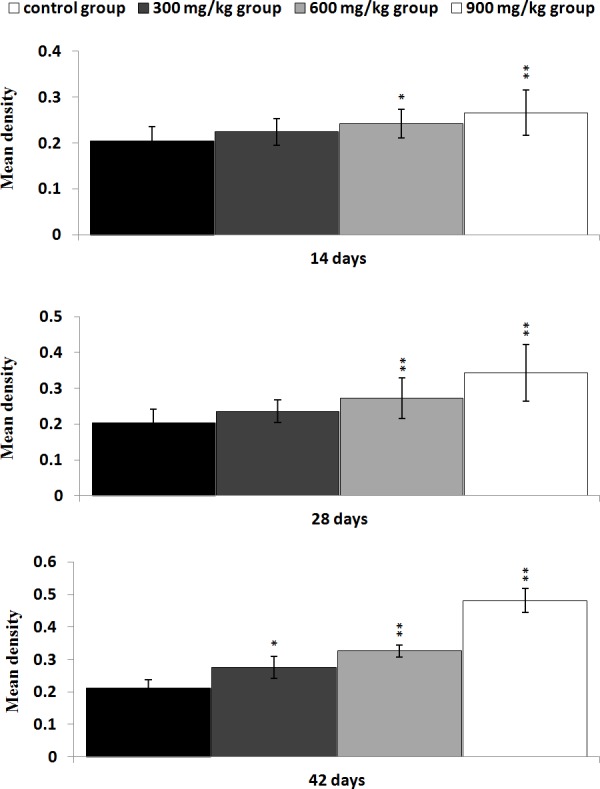
Changes of the mean density of NF-κB protein expression in the kidney Data are presented with the mean ± standard deviation (n=5×5) *P<0.05, compared with the control group **P<0.01, compared with the control group.

The NF-κB protein expression levels were significantly higher (*P* < 0.05 or *P* < 0.01) in the 600 mg/kg and 900 mg/kg groups at 14 and 28 days of age than those in the control group. The NF-κB protein expression levels were significantly increased (*P* < 0.05 or P < 0.01) in the three NiCl_2_-treated groups at 42 days of age when compared with those in the control group, as shown in Figures 4, 5, 6 and 7.

### Changes of inflammatory mediator mRNA expression levels in the kidney

**Figure 8:**
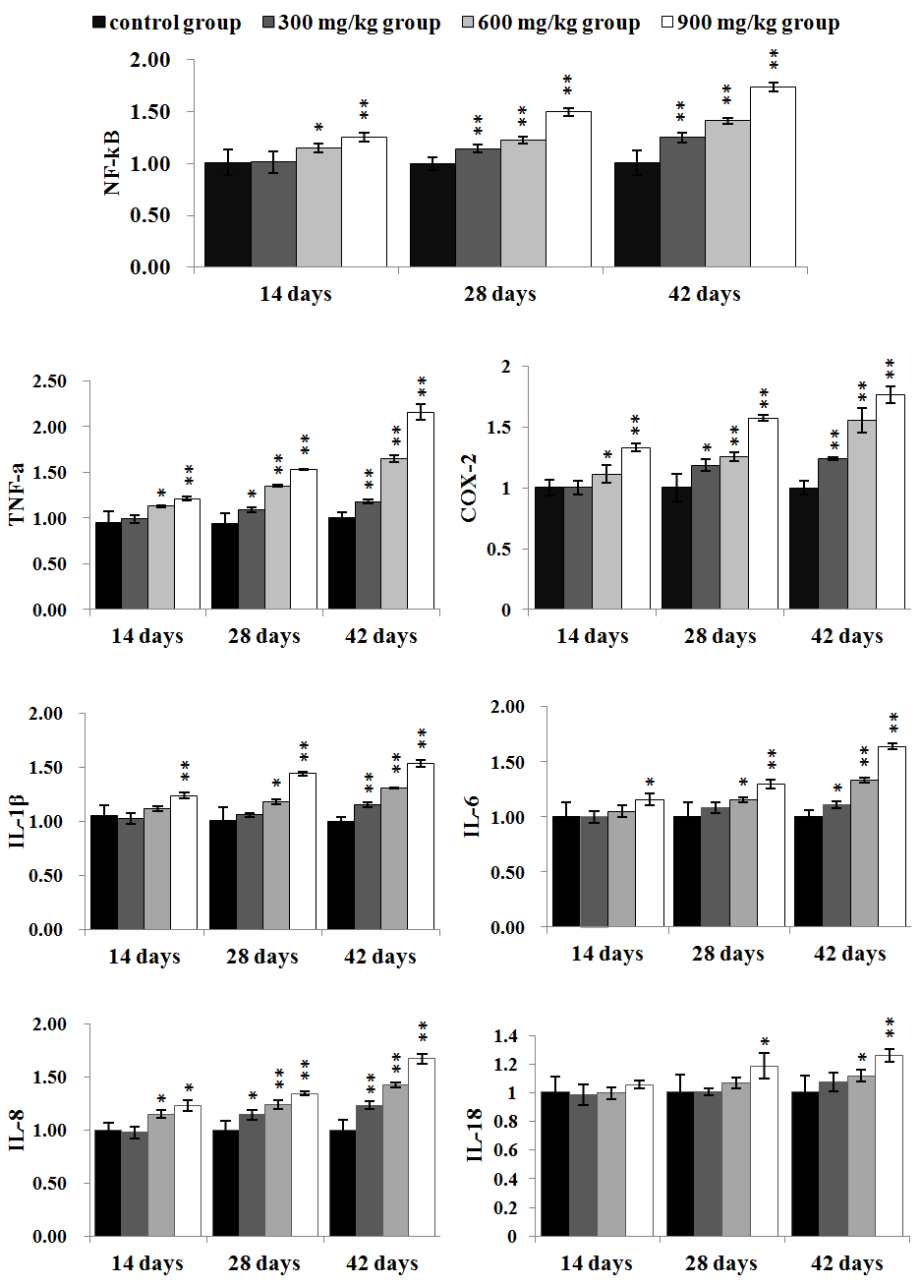
The mRNA expression levels of NF-κB, TNF-α, COX-2, IL-1β, IL-6, IL-8 and IL-18 in the kidney Data are presented with the mean ± standard deviation (n=5) *P<0.05, compared with the control group **P<0.01, compared with the control group.

The NF-κB, TNF-α, COX-2 and IL-8 mRNA expression levels were significantly higher (*P* < 0.05 or P < 0.01) in the 300 mg/kg group at 28 and 42 days of age, and in the 600 mg/kg, 900 mg/kg groups from 14 to 42 days of age than those in the control group. The IL-1β and IL-6 mRNA expression levels were significantly increased (*P* < 0.05 or *P* < 0.01) in the 900 mg/kg group at 14 days of age, and in the 300 mg/kg group at 42 days of age, and in the 600 mg/kg, 900 mg/kg groups at 28 and 42 days of age when compared with those in the control group. The IL-18 mRNA expression levels were significantly increased (*P* < 0.05 or *P* < 0.01) in the 900 mg/kg group at 28 days of age, and in the 600 mg/kg and 900 mg/kg groups at 42 days of age in comparison with those in the control group. The results were shown in Figure 8.

**Figure 9:**
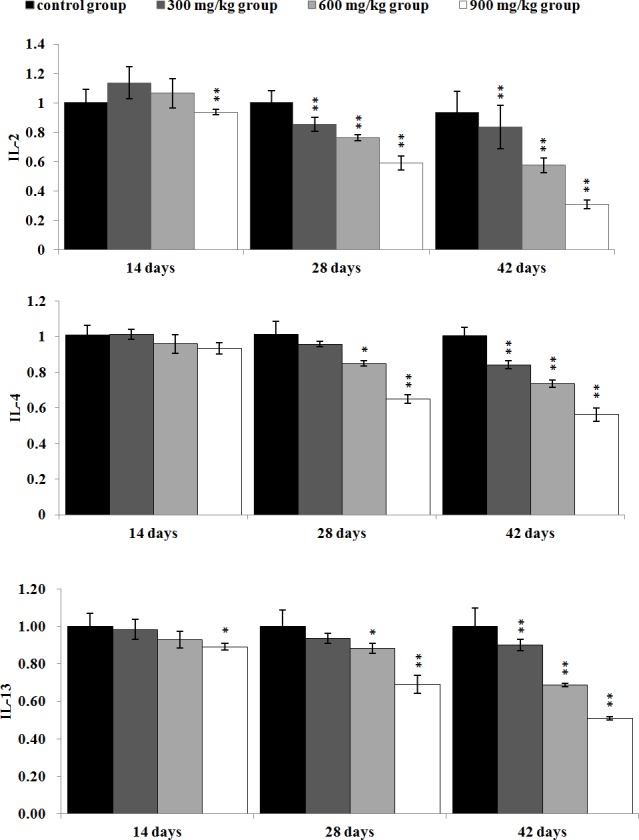
The mRNA expression levels of IL-2, IL-4 and IL-13 in the kidney Data are presented with the mean ± standard deviation (n=5) *P<0.05, compared with the control group **P<0.01, compared with the control group.

In Figure 9, the IL-2 mRNA expression levels were significantly lower (*P* < 0.05 or *P* < 0.01) in the 300 mg/kg, 600 mg/kg and 900 mg/kg from 28 to 42 days of age than those in the control group. The IL-4 and IL-13mRNA expression levels were significantly decreased (*P* < 0.05 or P < 0.01) in the 600 mg/kg and 900 mg/kg group at 28 days of age, and in the 300 mg/kg, 600 mg/kg, 900 mg/kg groups at 42 days of age in comparison with those in the control group. The IL-13 mRNA expression levels were significantly reduced (*P* < 0.05 or *P* < 0.01) in the 900 mg/kg group at 14 days of age.

### Changes of the renal function parameters

The Na^+^/K^+^-ATPase and Ca^2+^-ATPase activities were significantly lower (*P* < 0.05 or *P* < 0.01) in the 900 mg/kg group at 14 days of age and in the 300 mg/kg, 600 mg/kg and 900 mg/kg groups at 28 and 42 days of age than those in the control group, except the Na^+^/K^+^-ATPase and Ca^2+^-ATPase activities in the 300 mg/kg group at 28 days of age. The LDH activities were significantly decreased (*P* < 0.05 or *P* < 0.01) in the 900 mg/kg group at 14 days of age, and in the 600 mg/kg, 900 mg/kg groups at 28 and 42 days of age. The SDH and ACP activities were significantly lower (*P* < 0.05 or *P* < 0.01) in the 600 mg/kg and 900 mg/kg groups from 14 to 42 days of age than those in the control group.

The AKP activities were significantly higher (*P* < 0.05 or *P* < 0.01) in the 600 mg/kg group at 42 days of age, and in the 900 mg/kg groups from 14 to 42 days of age than those in the control group.

**Figure 10:**
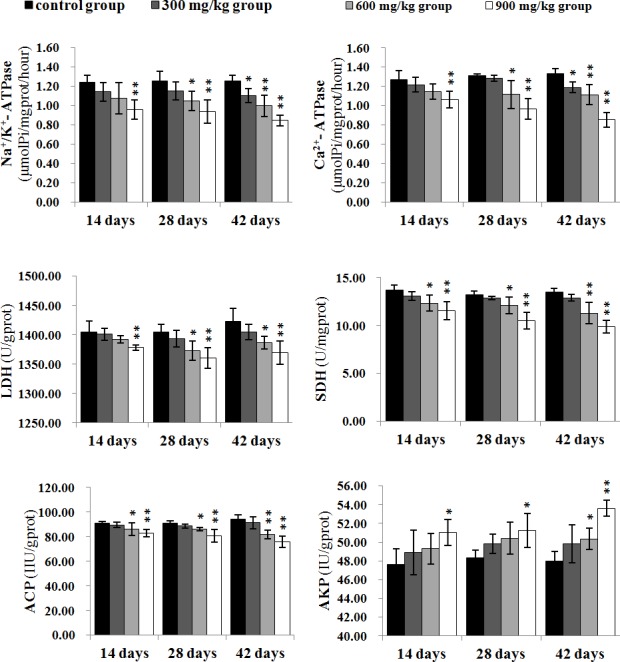
The changes of Na+/K+-ATPase, Ca2+-ATPase, LDH, SDH, ACP and AKP activities in the kidney Data are presented with the mean ± standard deviation (n=5) *P<0.05, compared with the control group **P<0.01, compared with the control group.

The results were shown in Figure 10.

### Changes of Ni contents in the kidney

**Figure 11:**
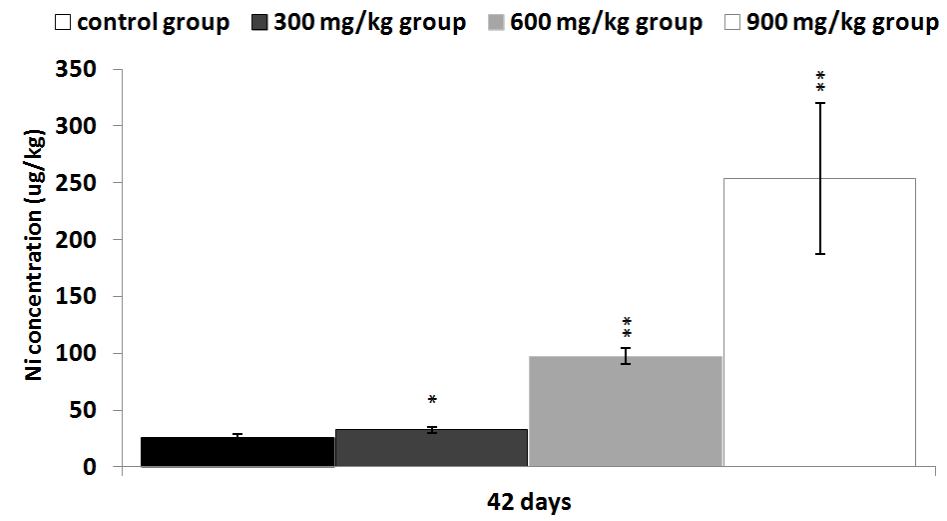
Changes of Ni contents in the kidney at 42 days Data are presented with the mean ± standard deviation (n=5) *P<0.05, compared with the control group **P<0.01, compared with the control group.

The Ni contents were increased in the three NiCl_2_-treated groups at 42 days of age in comparison with those in the control group (*P* < 0.05 or *P* < 0.01), as shown in Figure 11.

## DISCUSSION

This study focuses on NiCl_2_-induced renal inflammatory responses in the kidney. The results showed that NiCl_2_ enhanced transcription factor NF-κB and TNF-a, COX-2, IL-1β, IL-6, IL-8 and IL-18 mRNA expression levels, and NF-κB protein expression levels in the kidney. Our results are consistent with the results of Brant [24] in which NiSO_4_ increases COX-2 mRNA and protein expression levels in the lung. It has been reported that nickel hydroxide NPs increases the mRNA levels of TNF-α and IL-6 in the lung, spleen and heart of mouse [25]. Morimoto et al. [26] have reported that IL-1β protein expression levels in lung of rats are increased when exposured to NiONPs. Nickel subsulfide (Ni_2_S_3_) significantly increases IL-8 protein and mRNA expression levels in the human airway epithelial cells [27]. Transcription factor NF-κB, as a critical intracellular mediator of the inflammatory cascade, increases the expression of numerous genes including pro-inflammatory mediators such as TNF-α, COX-2, iNOS, IL-1β, IL-6, IL-8 and IL-18 that are correlated with the inflammatory response during experimental kidney injury [28]. Our results indicate that NiCl_2_ amplifies pro-inflammatory mediator secretion by activating the NF-κB signaling pathway, which is in agreement with the results obtained by Capasso et al. [10]. Also, the similar result demonstrates that nickel nitrate [Ni(NO_3_)_2_] significantly activates NF-κB pathway, and increases NF-κB and IL-8 protein expression levels in the THP-1 monocytic cells [29].

At the same time, NiCl_2_ reduced IL-2, IL-4 and IL-13 mRNA expression levels in the present study, which is consistent with the results that NiCl_2_ decreases IL-2, IL-4 and IL-13 mRNA expression in T cells [30]. Our previous studies have reported that NiCl_2_ decreased IL-2 expression in the thymus, intestinal mucosa, cecal tonsil and spleen [17, 31, 32]. IL-2, IL-4 and IL-13 are the main anti-inflammatory mediators and can inhibit pro-inflammatory mediator production [33, 34]. The reduction of IL-2, IL-4, and IL-13 mRNA expression may contribute to suppress anti-inflammatory status and to stimulate pro-inflammatory status.

**Figure 12:**
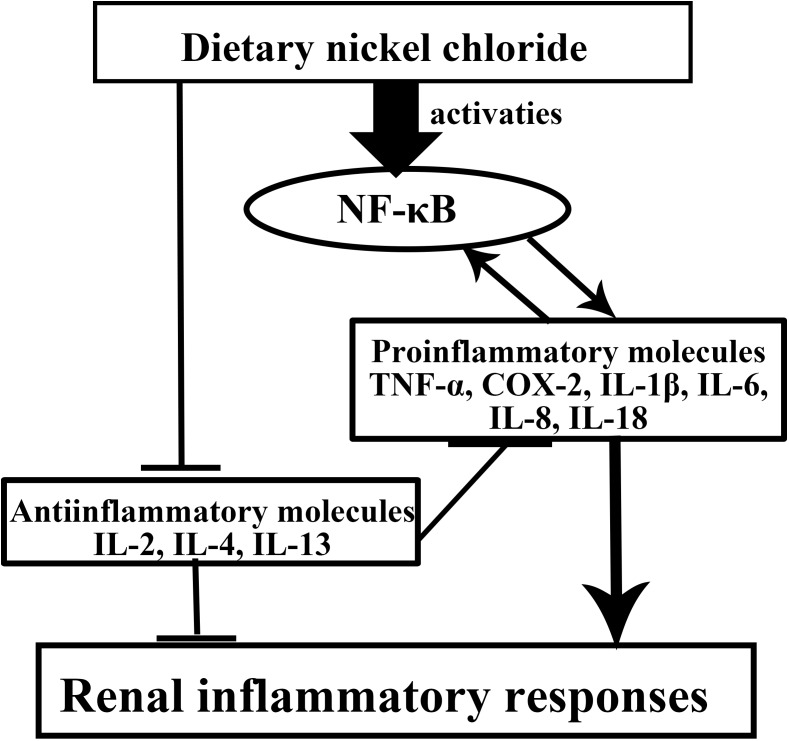
Mechanisms of NiCl2-cauced renal inflammatory responses Dietary NiCl_2_ in excess of 300 mg/kg induces increase of the pro-inflammatory mediator mRNA expression levels via NF-κB pathway and decrease of the anti-inflammatory mediator mRNA expression levels, which causes inflammatory responses in the kidney.

According to the results of present study and above-mentioned discussion, the mechanisms of NiCl_2_-caused renal inflammatory responses are summarized in Figure 12.

The imbalance between the production of pro-inflammatory and anti-inflammatory mediators can cause renal inflammatory responses, which impairs the renal function and tissue structure. In this study, NiCl_2_ induced histopathological injury, which is consistent with the alteration of renal function parameters including the elevation of AKP activity and the reduction of Na^+^/K^+^-ATPase, Ca^2+^-ATPase, LDH, SDH and ACP activities in the kidney. Tyagi et al. [21] have also reported that NiCl_2_ cause tubular swelling in the kidney of rat. The decrease and increase in the above-mentioned six enzyme activities caused by NiCl_2_ imply that the renal function is reduced or impaired.

The inflammatory responses, histopathological injury and the alteration of renal function parameters are consistent with Ni accumulation in the kidney (Figure 11), indicating that the Ni accumulation is main or/and direct reason of the renal injury.

In conclusion, dietary NiCl_2_ in excess of 300 mg/kg increases mRNA expression levels of the pro-inflammatory mediators including TNF-a, COX-2, IL-1β, IL-6, IL-8 and IL-18 via NF-κB activation, and decreases mRNA expression levels of anti-inflammatory mediators including IL-2, IL-4, and IL-13, which leads to renal inflammatory responses. This study demonstrates that activation of NF-κB pathway and reduction of anti-inflammatory mediator expression are main mechanisms of NiCl_2_-caused renal inflammatory responses. Concurrently, NiCl_2_ induces histopathological injury and decreases Na^+^/K^+^-ATPase, Ca^2+^-ATPase, LDH, SDH and ACP activities, and increases AKP activity in the kidney. The above-mentioned results represent reduction or impairment of the renal function.

## MATERIALS AND METHODS

### Animals and treatment

Two hundred and eighty one-day-old healthy broilers were divided into four groups. There were seventy broilers in each group. Broilers were housed in cages with electrical heaters, and provided with water as well as under-mentioned experimental diets *ad libitum* for 42 days. The commercial broilers’ growth cycle is about 42 days, and then they will be put into use for consumption. In this period they grow rapidly and a lot of diet will be consumed, and broilers will easily affected by diet containing metal pollutants (such as Ni). The aim of our study is to evaluate the effect of dietary NiCl_2_ on the broilers in the period of growth.

To observe the time-dependent dynamic change, we chose three time points (14, 28 and 42 days of age) for examining histopathological injury, renal function parameter changes, and inflammatory mediator protein expression and mRNA expression levels.

In this study, a corn-soybean basal diet formulated by the National Research Council [35] was the control diet. NiCl_2_ (NiCl_2_·6H_2_O, ChengDu Kelong Chemical Co., Ltd., Chengdu, China) was mixed into the corn-soybean basal diet to produce the experimental diets containing 300, 600 and 900 mg/kg NiCl_2_, respectively.

The basis of doses (300, 600 and 900 mg/kg NiCl_2_) selection: Ling and Leach reported that dietary NiCl_2_ concentrations of 300 mg/kg and over resulted in significant reduction in growth rate. Mortality and anemia were observed in chicks receiving 1100 mg/kg nickel [16]. Weber and Reid found a significant growth reduction at 700 mg/kg NiSO_4_ and nickel acetate and over [36]. Chicks fed more than 250-300 mg/kg Ni in the diet exhibited depressed growth and reduced feed intake [37]. Bersenyi et al. [38] reported that supplementation of 500 mg/kg NiCl_2_ decreased weight gain (by 10%), feed intake (by 4%) and worse FCE (by 5%) in growing broiler cockerels. According to the above-mentioned research results and our preliminary experiment, we chose the doses of 300, 600 and 900mg/kg NiCl_2_ in this study for observing the does-dependent changes.

Our experiments involving the use of broilers and all experimental procedures were approved by Animal Care and Use Committee, Sichuan Agricultural University.

### Histopathological examination of kidney

Five chickens in each group were humanely killed at 14, 28 and 42 days of age. Kidneys were removed, fixed in 4% paraformaldehyde, dehydrated in ethanol and embedded in paraffin. Serial slices at 5 μm thickness were prepared and stained with haematoxylin and eosin (H&E), and examined by light microscopy.

### Determination of the NF-κB protein expression by immunohistochemistry

Five chickens in each group were humanely sacrificed for gross examination at 14, 28 and 42 days of age. Kidneys were collected and fixed in 4% paraformaldehyde, dehydrated in ethanol and embedded in paraffin.

As described by Wu et al. [18], renal slices were dewaxed in xylene, rehydrated through a graded series of ethanol solutions, washed in distilled water and PBS and endogenous peroxidase activity was blocked by incubation with 3% H_2_O_2_ in methanol for 15 min. The sections were subjected to antigen retrieval procedure by microwaving in 0.01 M sodium citrate buffer pH 6.0. Additional washing in PBS was performed before 30 min of incubation at 37°C in 10% normal goat serum (Boster, Wuhang, China). The slices were incubated overnight at 4°C with anti-NF-κB (phospho S536) (1:100) antibody (bs-0465R, Bioss, Beijing, China). After washing in PBS, the slices were exposed to 1% biotinylated goat anti-mouse IgG secondary antibody (Boster, Wuhang, China) for 1 h at 37°C, and then incubated with strept avidin-biotin complex (SABC; Boster, Wuhang, China) for 30 min at 37°C. To visualize the immunoreaction, sections were immersed in diaminobenzidine hydrochloride (DAB; Boster, Wuhang, China). The slices were monitored microscopically and stopped by immersion in distilled water, as soon as brown staining was visible. Slices were lightly counterstained with hematoxylin, dehydrated in ethanol, cleared in xylene and mounted. In the slices, the NF-κB protein was brown-stain.

The NF-κB protein expression was counted using a computer-supported imaging system connected to a light microscope (OlympusAX70) with an objective magnification of ×400. The intensity of staining for each protein was quantified using Image-pro Plus 5.1 (USA). Each group was measured five sections and each section was measured five visions and averaged.

### Determination of the inflammatory mediator mRNA expression by qRT-PCR

The kidneys from five broilers in each group at 14, 28, and 42 days of age were stored in liquid nitrogen, and then the kidney samples were homogenized with liquid nitrogen using a mortar and pestle.

As described Wu et al. [18], total RNA was extracted from the powder of kidney using RNAiso Plus (9108/9109, Takara, Japan). The cDNA was synthesized using a Prim-Script™ RT reagent Kit (RR047A, Takara, Japan) according to the manufacturer's instructions. The cDNA was used as a template for qRT-PCR analysis. Sequences for primers were obtained from Genbank and NCBI. Primers were designed using Primer 5 and synthesized at Takara (Dalian, China) (Table 1).

**Table 1:** A list of primers in qRT-PCR analysis of mRNA expression of the inflammatory mediators

Gene symbol	Accession number	Primer	Primer sequence(5′-3′)	Product size	Tm (°C)
NF-κB	NM205134	Forward Reverse	CTGAAACTACTGATTGCTGCTGGA GCTATGTGAAGAGGCGTTGTGC	179bp	62
TNF-α	NM204267	Forward Reverse	CCCCTACCCTGTCCCACAA TGAGTACTGCGGAGGGTTCAT	100bp	58
COX-2	NM001167718	Forward Reverse	CTTAAATTGAGACTTCGCAAGGATG TGGGACCAAGCCAAACACCT	165bp	62
IL-1β	Y15006	Forward Reverse	CAGCCTCAGCGAAGAGACCTT CACTGTGGTGTGCTCAGAATCC	106bp	60
IL-2	AF000631	Forward Reverse	TCTGGGACCACTGTATGCTCT ACACCAGTGGGAAACAGTATCA	138bp	60
IL-4	AJ621249	Forward Reverse	ACCCAGGGCATCCAGAAG CAGTGCCGGCAAGAAGTT	176bp	59
IL-6	AJ309540	Forward Reverse	AATCCCTCCTCGCCAATCTG GCCCTCACGGTCTTCTCCATA	106bp	60
IL-8	HM179639	Forward Reverse	CTGGCCCTCCTCCTGGTT GCAGCTCATTCCCCATCTTTAC	105bp	60
IL-13	AJ621250	Forward Reverse	AGTGCTGGACAACATGACCGA GCAAGAAGTTCCGCAGGTAGAT	128bp	60
IL-18	AJ277865	Forward Reverse	TAGCCAGTTGCTTGTGGTTCG TCTTATCTTCTACCTGGACGCTGA	170bp	60
β-actin	L08165	Forward Reverse	TGCTGTGTTCCCATCTATCG TTGGTGACAATACCGTGTTCA	178bp	62

qRT-PCR was performed on a Thermal Cycler (C1000, BIO RAD, USA). The total reaction volume was 25 μL: 2 μL cDNA, 12.5 μL of SYBR^®^ Premix Ex Taq™ II (DRR820A, Takara, Japan), 1μL of each primer (10 μM) and 8.5 μL of RNAase-free water. Cycle parameters were as follows: activation at 95°C for 3 min, 44 cycles of 95°C for10 s, Tm of a specific primer pair for 30 s, and then 95°C for 10 s, 72°C for 10 s. The melting curve analysis showed only one peak for each PCR product. Electrophoresis was performed with the PCR products to verify primer specificity and product purity.

Chicken β-actin expression as a housekeeping gene was used as an internal reference. Gene expression values of control group at 14, 28 and 42 days of age were used for gene expression calibration. Data from the qRT-PCR were analyzed using the 2^−ΔΔCT^ method [39].

### Determination of the renal function parameters

The renal function parameters were determined according to the method described by Wu et al. [18]. After five broilers in each group were humanely killed at 14, 28, and 42 days of age, kidneys were immediately removed, weighed and homogenized in cold physiological saline to give 10% homogenate (using glass homogenizer), and centrifuged at 3,500×g at 4°C for 10 min. The supernatant were finally collected. After determining the amount of total protein in the supernatant by the method of Bradford [40], the activity of Na^+^/K^+^-ATPase, Ca^2+^-ATPase, LDH, SDH, AKP, and ACP in the supernatant were detected by biochemical methods following the instruction of the reagent kits (ATPase, A016-2; LDH, A020-2; SDH, A022; AKP, A059-2; ACP, A060-2; total protein, A045-2, purchased from Nanjing Jiancheng Bioengineering Institute of China, Nanjing, China). Their absorbance were measured at 660, 440, 600, 520, 520 and 590 nm, respectively, using a microtiter plate reader (Thermo, Varioskan Flash, USA).

### Determination of the renal Ni contents by graphite furnace atomic absorption spectrometry (GFAAS)

After five broilers in each group were humanely killed at 42 days of age, kidneys were immediately removed, weighed, dried, and collected for determination of Ni contents.

Ni contents in the kidney were measured by GFAAS according to the reference [41].

### Statistical analysis

The significance of difference among four groups was analyzed by variance analysis, and results presented as mean ± standard deviation ($\bar{X}$ ± SD). The variation was measured by one-way analysis of variance (ANOVA) test of SPSS 16.0 for windows. Statistical significance was considered at *P* < 0.05.

## References

[1] Cempel M, Nikel G (2006). Nickel: A Review of Its Sources and Environmental Toxicology. Pol J Environ Stud.

[2] Lu H, Shi X, Costa M, Huang C (2005). Carcinogenic effect of nickel compounds. Mol Cell Biochem.

[3] Pasanen K, Pukkala E, Turunen AW, Patama T, Jussila I, Makkonen S, Salonen RO, Verkasalo PK (2012). Mortality among population with exposure to industrial air pollution containing nickel and other toxic metals. J Occup Environ Med.

[4] Alarifi S, Ali D, Alakhtani S, Al Suhaibani ES, Al-Qahtani AA (2014). Reactive oxygen species-mediated DNA damage and apoptosis in human skin epidermal cells after exposure to nickel nanoparticles. Biol Trace Elem Res.

[5] Das KK, Das SN, Dhundasi SA (2008). Nickel, its adverse health effects & oxidative stress. Indian J Med Res.

[6] Salnikow K, Costa M (1999). Epigenetic mechanisms of nickel carcinogenesis. J Environ Pathol Toxicol Oncol.

[7] Costa M, Klein CB (1999). Nickel carcinogenesis, mutation, epigenetics, or selection. Environ Health Persp.

[8] Goodman JE, Prueitt RL, Dodge DG, Thakali S (2009). Carcinogenicity assessment of water-soluble nickel compounds. Crit Rev Toxicol.

[9] Zheng GH, Liu CM, Sun JM, Feng ZJ, Cheng C (2014). Nickel-induced oxidative stress and apoptosis in Carassius auratus liver by JNK pathway. Aquat Toxicol.

[10] Capasso L, Camatini M, Gualtieri M (2014). Nickel oxide nanoparticles induce inflammation and genotoxic effect in lung epithelial cells. Toxicol Lett.

[11] Brant KA, Fabisiak JP (2009). Nickel and the microbial toxin, MALP-2, stimulate proangiogenic mediators from human lung fibroblasts via a HIF-1alpha and COX-2-mediated pathway. Toxicol Sci.

[12] Hattiwale SH, Saha S, Yendigeri SM, Jargar JG, Dhundasi SA, Das KK (2013). Protective effect of L-ascorbic acid on nickel induced pulmonary nitrosative stress in male albino rats. Biometals : an international journal on the role of metal ions in biology, biochemistry, and medicine.

[13] Cai T, Li X, Ding J, Luo W, Li J, Huang C (2011). A cross-talk between NFAT and NF-κB pathways is crucial for nickel-induced COX-2 expression in Beas-2B cells. Curr cancer drug tar.

[14] Tak PP, Firestein GS (2001). NF-kappaB: a key role in inflammatory diseases. The Journal of clinical investigation.

[15] Morimoto Y, Ogami A, Todoroki M, Yamamoto M, Murakami M, Hirohashi M, Oyabu T, Myojo T, Nishi K-I, Kadoya C (2010). Expression of inflammation-related cytokines following intratracheal instillation of nickel oxide nanoparticles. Nanotoxicology.

[16] Ling J, Leach R (1979). Studies on nickel metabolism: interaction with other mineral elements. Poultry Sci.

[17] Wu B, Cui H, Peng X, Fang J, Zuo Z, Deng J, Wang X, Huang J (2014). Toxicological effects of nickel chloride on the cytokine mRNA expression and protein levels in intestinal mucosal immunity of broilers. Environ Toxicol.

[18] Wu B, Cui H, Peng X, Fang J, Zuo Z, Deng J, Huang J (2014). Dietary nickel chloride induces oxidative stress, apoptosis and alters Bax/Bcl-2 and caspase-3 mRNA expression in the cecal tonsil of broilers. Food Chem Toxicol.

[19] Huang J, Cui H, Peng X, Fang J, Zuo Z, Deng J, Wu B (2013). The Association between Splenocyte Apoptosis and Alterations of Bax, Bcl-2 and Caspase-3 mRNA Expression, and Oxidative Stress Induced by Dietary Nickel Chloride in Broilers. Int J Environ Res Public Health.

[20] Guo H, Wu B, Cui H, Peng X, Fang J, Zuo Z, Deng J, Wang X, Deng J, Yin S, Li J, Tang K (2014). NiCl2-down-regulated antioxidant enzyme mRNA expression causes oxidative damage in the broiler(‘)s kidney. Biol Trace Elem Res.

[21] Tyagi R, Rana P, Gupta M, Khan AR, Bhatnagar D, Bhalla PJ, Chaturvedi S, Tripathi RP, Khushu S (2013). Differential biochemical response of rat kidney towards low and high doses of NiCl2 as revealed by NMR spectroscopy. J Appl Toxicol.

[22] Jalbani N, Soylak M (2015). Ligandless ultrasonic-assisted and ionic liquid-based dispersive liquid-liquid microextraction of copper, nickel and lead in different food samples. Food Chem.

[23] Denkhausa E SK (2002). Nickel essentiality, toxicity, and carcinogenicity. Crit Rev Oncol Hemat.

[24] Brant KA, Fabisiak JP (2008). Nickel alterations of TLR2-dependent chemokine profiles in lung fibroblasts are mediated by COX-2. Am J Resp Cell Mol.

[25] Kang GS, Gillespie PA, Gunnison A, Moreira AL, Tchou-Wong KM, Chen LC (2011). Long-term inhalation exposure to nickel nanoparticles exacerbated atherosclerosis in a susceptible mouse model. Environ Health Persp.

[26] Morimoto Y, Ogami A, Todoroki M, Yamamoto M, Murakami M, Hirohashi M, Oyabu T, Myojo T, Nishi K, Kadoya C, Yamasaki S, Nagatomo H, Fujita K, Endoh S, Uchida K, Yamamoto K (2010). Expression of inflammation-related cytokines following intratracheal instillation of nickel oxide nanoparticles. Nanotoxicology.

[27] Barchowsky A, Soucy NV, O'Hara KA, Hwa J, Noreault TL, Andrew AS (2002). A novel pathway for nickel-induced interleukin-8 expression. J Biol Chem.

[28] Sanz AB, Sanchez-Nino MD, Ramos AM, Moreno JA, Santamaria B, Ruiz-Ortega M, Egido J, Ortiz A (2010). NF-kappaB in renal inflammation. J Am Soc Nephrol.

[29] Freitas M, Fernandes E (2011). Zinc, cadmium and nickel increase the activation of NF-κB and the release of cytokines from THP-1 monocytic cells. Metallomics : integrated biometal science.

[30] Saito R, Hirakawa S, Ohara H, Yasuda M, Yamazaki T, Nishii S, Aiba S (2011). Nickel differentially regulates NFAT and NF-kappaB activation in T cell signaling. Toxicol Appl Pharmacol.

[31] Huang J, Cui H, Peng X, Fang J, Zuo Z, Deng J, Wang X, Wu B (2014). Effect of dietary nickel chloride on splenic immune function in broilers. Biol Trace Elem Res.

[32] Tang K, Guo H, Deng J, Cui H, Peng X, Fang J, Zuo Z, Wang X, Wu B, Li J, Yin S (2015). Inhibitive Effects of Nickel Chloride (NiCl2) on Thymocytes. Biol Trace Elem Res.

[33] Dinarello CA (2000). Proinflammatory cytokines. Chest.

[34] Coppo R, Amore A (2004). New perspectives in treatment of glomerulonephritis. Pediatr Nephrol.

[35] NRC (1994). Nutrient Requirements of Poultry.

[36] Weber CW, Reid BL (1968). Nickel toxicity in growing chicks. J Nutr.

[37] Szilagyi M, Szentmihalyi S, Anke M (1981). Changes in some of the biochemical parameters in Ni and Mo deficient animals [goat, sheep, pig, chicken, rat].

[38] Bersényi A, Fekete SG, Szilágyi M, Berta E, Zöldág L, Glávits R (2004). Effects of nickel supply on the fattening performance and several biochemical parameters of broiler chickens and rabbits. Acta Vet Hungarica.

[39] Livak KJ, Schmittgen TD (2001). Analysis of relative gene expression data using real-time quantitative PCR and the 2(-Delta Delta C(T)) method. Methods.

[40] Bradford MM (1976). A rapid and sensitive method for the quantitation of microgram quantities of protein utilizing the principle of protein-dye binding. Anal Biochem.

[41] Jihen el H, Imed M, Fatima H, Abdelhamid K (2008). Protective effects of selenium (Se) and zinc (Zn) on cadmium (Cd) toxicity in the liver and kidney of the rat: histology and Cd accumulation. Food Chem Toxicol.

